# Genome Sequence of *Plesiomonas shigelloides* Strain 302-73 (Serotype O1)

**DOI:** 10.1128/genomeA.00404-13

**Published:** 2013-06-27

**Authors:** Núria Piqué, Eleonora Aquilini, Tyler Alioto, David Miñana-Galbis, Juan M. Tomás

**Affiliations:** Departamento de Microbiología y Parasitología Sanitarias, Facultad de Farmacia, Universitat de Barcelona, Barcelona, Spaina; Departamento de Microbiología, Facultad de Biología, Universitat de Barcelona, Barcelona, Spainb; Centre Nacional d’Anàlisi Genòmica (CNAG), Parc Científic de Barcelona, Barcelona, Spainc

## Abstract

*Plesiomonas shigelloides*, the only species of the genus, is an emergent pathogenic bacterium associated with human diarrheal and extraintestinal disease. We present the whole-genome sequence analysis of the representative strain for the O1 serotype (strain 302-73), providing a tool for studying bacterial outbreaks, virulence factors, and accurate diagnostic methods.

## GENOME ANNOUNCEMENT

*Plesiomonas shigelloides*, the only species of the genus, is a Gram-negative, flagellated, rod-shaped, ubiquitous, and facultative anaerobic bacterium that has been isolated from sources, such as freshwater, surface water, and many wild and domestic animals (1). *P. shigelloides* is associated with diarrheal disease in humans (2), as found in several types of gastroenteritis, including acute secretory gastroenteritis (3), an invasive shigellosis-like disease (4), and a cholera-like illness (5). Extraintestinal infections, such as meningitis, bacteremia (6), and pseudoappendicitis (7), are also associated with *P. shigelloides* infection.

It is considered an emergent bacterial pathogen, as there has been an increasing number of reports describing *P. shigelloides* infections in recent years (8) (about 5% of traveler’s diarrhea is produced by *P. shigelloides* infection). To date, however, the lack of routine analysis for *P. shigelloides* in cases of gastroenteritis leads to only sporadic and occasional identification of this bacterium (8, 9). Therefore, the greatest challenge is to develop a rapid, early, and accurate diagnostic method for the detection of *P. shigelloides* (8).

The bacterium remained within the family *Vibrionaceae* until molecular studies carried out by Martínez-Murcia et al. (10) indicated that *P. shigelloides* is phylogenetically related to the genus *Proteus*, although it lately has been reported to be more related to the organism *Edwardsiella tarda* (11). The genus *Plesiomonas* has been reclassified under the family *Enterobacteriaceae* and is the only oxidase-positive member of this family (12). Compared to other phenotypic methods, serology has been used more successfully for distinguishing different strains of *P. shigelloides*. There are mainly two major serotyping schemes, which are based on somatic (O) and flagellar (H) antigens. At the present moment, 102 somatic antigens and 51 flagellar antigens have been recognized (13).

*P. shigelloides* strain 302-73, isolated in Japan (14), is the representative strain for the O1 serotype, whose complete lipopolysaccharide (LPS) has been chemically characterized (15, 16). For the first time, we have obtained the whole-genome sequence of *P. shigelloides* 302-73. We think this information will contribute to the study of virulence factors and in the development of new accurate diagnostic methods.

For whole-genome sequencing, two runs of 8-kb paired-end sequencing using the GS FLX system (Roche Diagnostics) produced 80× genome coverage. Read assembly with the Newbler software (Roche Diagnostics) resulted in 13 large scaffolds, two of which account for >90% of the assembly (N_90_, 1.76 Gb), and a total of 389 contigs >250 bp in length (N_50_, 86.6 kb; N_90_, 13.5 kb). Genome annotation was performed automatically via the Rapid Annotations using Subsystems Technology (RAST) server (17) and also by the NCBI Prokaryotic Genomes Annotation Pipeline (PGAAP). The assembled genome of *P. shigelloides* comprises a single circular chromosome (3.9 Mbp, 51.2% G+C content), with 3,285 coding DNA sequences (CDSs), 7 rRNAs, and 96 tRNA sequences (according to the RAST annotation).

### Nucleotide sequence accession numbers.

The draft genome sequences for *P. shigelloides* 302-73 have been deposited at DDBJ/EMBL/GenBank under the accession no. AQQO00000000 (BioProject PRJNA196338). The version described in this paper is the first version, accession no. AQQO01000000.

## References

[1] Wong TY Tsui HY So MK Lai JY Lai ST Tse CW Ng TK 2000 Plesiomonas shigelloides infection in Hong Kong: retrospective study of 167 laboratory-confirmed cases. Hong Kong Med. J. 6:375–380 11177159

[2] Brenden RA Miller MA Janda JM 1988 Clinical disease spectrum and pathogenic factors associated with *Plesiomonas shigelloides* infections in humans. Rev. Infect. Dis. 10:303–316 3287561 10.1093/clinids/10.2.303

[3] Mandal BK Whale K Morson BC 1982 Acute colitis due to *Plesiomonas shigelloides*. Br. Med. J. (Clin. Res. Ed.) 285:1539–1540 10.1136/bmj.285.6354.1539-a PMC1500530 6814640

[4] McNeeley D Ivy P Craft JC Cohen I 1984 *Plesiomonas*: biology of the organism and diseases in children. Pediatr. Infect. Dis. 3:176–181 6374631

[5] Tsukamoto T Kinoshita Y Shimada T Sakazaki R 1978 Two epidemics of diarrhoeal disease possibly caused by *Plesiomonas shigelloides*. J. Hyg. (Lond.) 80:275–280 632567 10.1017/s0022172400053638 PMC2130002

[6] Billiet J Kuypers S Van Lierde S Verhaegen J 1989 Plesiomonas shigelloides meningitis and septicaemia in a neonate: report of a case and review of the literature. J. Infect. 19:267–271 2689523 10.1016/s0163-4453(89)90809-8

[7] Fischer K Chakraborty T Hof H Kirchner T Wamsler O 1988 Pseudoappendicitis caused by *Plesiomonas shigelloides*. J. Clin. Microbiol. 26:2675–2677 3230143 10.1128/jcm.26.12.2675-2677.1988 PMC266973

[8] Meng S Xu J Xiong Y Ye C 2012 Rapid and sensitive detection of *Plesiomonas shigelloides* by loop-mediated isothermal amplification of the *hugA* gene. PLoS One 7:e41978 23077478 10.1371/journal.pone.0041978 PMC3471923

[9] Chan SS Ng KC Lyon DJ Cheung WL Cheng AF Rainer TH 2003 Acute bacterial gastroenteritis: a study of adult patients with positive stool cultures treated in the emergency department. Emerg. Med. J. 20:335–338 12835343 10.1136/emj.20.4.335 PMC1726145

[10] Martinez-Murcia AJ Benlloch S Collins MD 1992 Phylogenetic interrelationships of members of the genera *Aeromonas* and *Plesiomonas* as determined by 16S ribosomal DNA sequencing: lack of congruence with results of DNA-DNA hybridizations. Int. J. Syst. Bacteriol. 42:412–421 1380289 10.1099/00207713-42-3-412

[11] Salerno A Delétoile A Lefevre M Ciznar I Krovacek K Grimont P Brisse S 2007 Recombining population structure of *Plesiomonas shigelloides* (*Enterobacteriaceae*) revealed by multilocus sequence typing. J. Bacteriol. 189:7808–7818 17693512 10.1128/JB.00796-07 PMC2168737

[12] Garrity GM Winters M Searles DB 2001 Taxonomic outline of the prokaryotic genera, p 13 *In* Garrity GM, Bergey’s manual of systematic bacteriology, 2nd ed. Springer-Verlag, New York, NY.

[13] Aldova E Shimada T 2000 New O and H antigens of the international antigenic scheme for *Plesiomonas shigelloides*. Folia Microbiol. 45:301–304 11347249 10.1007/BF02817550

[14] Shimada T Sakazaki R 1978 On the serology of *Plesiomonas shigelloides*. Jpn. J. Med. Sci. Biol. 31:135–142 682370 10.7883/yoken1952.31.135

[15] Pieretti G Corsaro MM Lanzetta R Parrilli M Canals R Merino S Tomás JM 2008 Structural studies of the O-chain polysaccharide from *Plesiomonas shigelloides* strain 302–73 (serotype O1). Eur. J. Org. Chem. 8:3149–3155

[16] Pieretti G Carillo S Lindner B Lanzetta R Parrilli M Jimenez N Regué M Tomás JM Corsaro MM 2010 The complete structure of the core of the LPS from *Plesiomonas shigelloides* 302–73 and the identification of its O-antigen biological repeating unit. Carbohydr. Res. 345:2523–2528 20933222 10.1016/j.carres.2010.09.007

[17] Aziz RK Bartels D Best AA DeJongh M Disz T Edwards RA Formsma K Gerdes S Glass EM Kubal M Meyer F Olsen GJ Olson R Osterman AL Overbeek RA McNeil LK Paarmann D Paczian T Parrello B Pusch GD Reich C Stevens R Vassieva O Vonstein V Wilke A Zagnitko O 2008 The RAST Server: rapid annotations using subsystems technology. BMC Genomics 9:75 18261238 10.1186/1471-2164-9-75 PMC2265698

